# An endoscopic triumph: transgastric balloon-anchored antegrade dilation via gastrostomy reopens a “no-pass” esophagus

**DOI:** 10.1055/a-2839-9338

**Published:** 2026-04-22

**Authors:** Xiaonan Shen, Ke Qi, Minmin Zhang

**Affiliations:** 1 Department of GastroenterologyRuijin Hospital Affiliated to Shanghai Jiao Tong University School of MedicineShanghaiChina

A 64-year-old man underwent right radical neck dissection, partial laryngopharyngectomy, pedicled myocutaneous flap reconstruction, and tracheostomy for right hypopharyngeal squamous cell carcinoma, followed by standard adjuvant chemoradiotherapy. Due to persistent dysphagia, esophagoscopy was performed and revealed no esophageal inlet, prompting percutaneous endoscopic gastrostomy (PEG) for enteral nutrition.

**Fig. 1:**
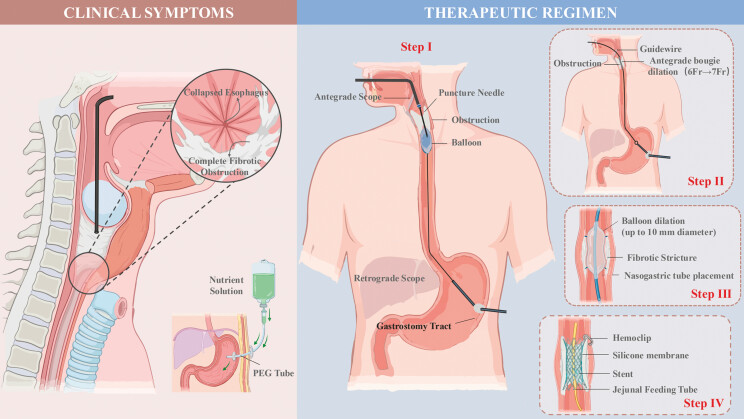
The cartoon of the whole procedure.

**Fig. 2:**
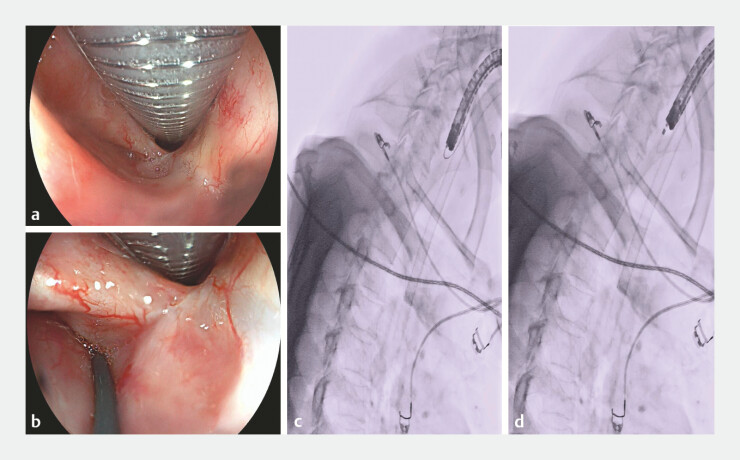
Complete esophageal obstruction. **a** Fibrotic scarring with mucosal convergence in the pharynx. **b–d** Guidewire advancement attempts (including with a 6 Fr dilator) failed.

For 4 years, the patient harbored a strong desire to resume oral intake. Supported by his family, he presented to our hospital. The whole procedure is shown in Fig. 1 . Gastroscopy demonstrated fibrotic scarring with mucosal convergence in the pharynx, with no patent lumen visible at either a left or a right esophageal inlet. Contrast injection confirmed the absence of esophageal passage. Guidewire advancement attempts (including with a 6Fr dilator) failed, suggesting complete esophageal obstruction ( Fig. 2 ).

**Fig. 3:**
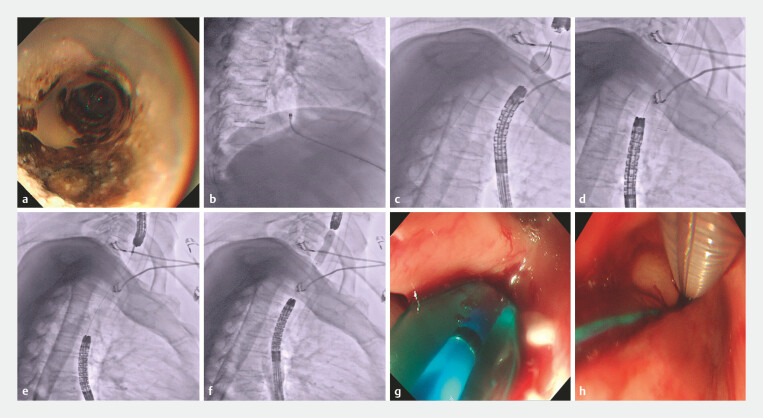
Establishing a food passage. **a** The PEG tube. **b-c** An endoscope through the gastrostomy tract into the gastric cavity and distal esophagus. A balloon was positioned in the proximal esophagus. Transgastric puncture into the balloon with endoscopic ultrasound guidance facilitated guidewire placement. **d** – **g** The endoscope was reinserted via the gastrostomy tract to snare the guidewire, allowing antegrade 6 Fr bougie dilation, gradually up to 7 Fr. Stepwise electrocautery incision and balloon dilation (up to 10 mm diameter) were performed. **h** Nasogastric tube placement.

Then, we removed the PEG tube and advanced the endoscope through the gastrostomy tract into the gastric cavity and distal esophagus. A balloon was positioned in the distal esophagus. Trans-esophageal puncture into the balloon with forward-viewer endoscopic ultrasound guidance facilitated guidewire placement. The endoscope was reinserted via the gastrostomy tract to snare the guidewire, allowing antegrade 6 Fr bougie dilation, gradually up to 7 Fr. Stepwise balloon dilation (up to 10 mm diameter) was performed, followed by nasogastric tube placement ( Fig. 3 ). Postoperative prednisone was administered to prevent the restenosis of esophagus. The patient continued PEG feeding.

**Fig. 4:**
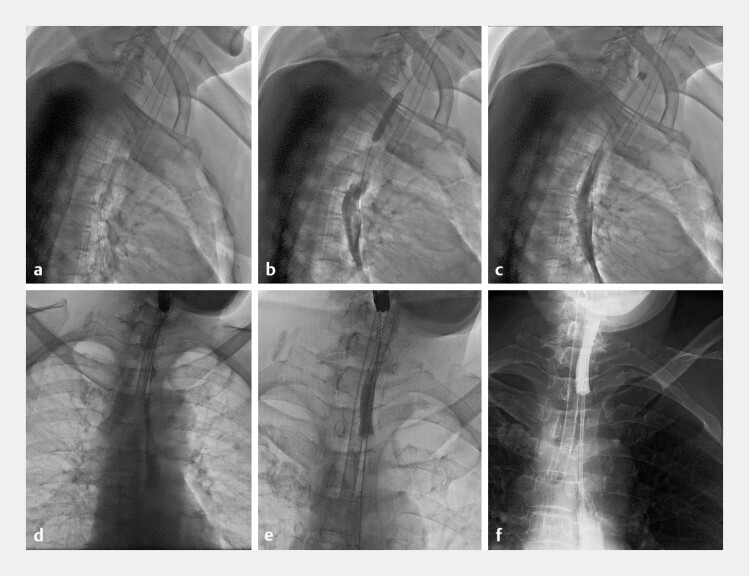
**a–e** The nasogastric tube was exchanged for a Cook covered biliary metal stent. **f** Subsequent jejunal feeding tube placement.

One month later, a nasogastric tube was exchanged for a Cook covered biliary metal stent, with subsequent jejunal feeding tube placement and clip fixation of the stent’s retrieval ring ( Fig. 4 ). After 2 months, the stent was removed, and the patient resumed oral feeding without complications.

This is the first case of endoscopic ultrasound-guided puncture via a gastrostomy sinus tract with balloon guided to establish a food passage in a patient with esophageal atresia, which holds significant importance for individuals with esophageal atresia aspiring to achieve oral intake ( Video 1 ).

This video is the first reported case of endoscopic ultrasound-guided puncture via a gastrostomy sinus tract with balloon guided to establish a food passage in a patient with esophageal atresia, which holds significant importance for individuals with esophageal atresia aspiring to achieve oral intake.Video 1

Endoscopy_UCTN_Code_TTT_1AO_2AZ

